# Effect of Copper Selenide Modification on the Conductivity of PA6, PA66, PAN, and PES Fibers

**DOI:** 10.3390/ma15124320

**Published:** 2022-06-18

**Authors:** Daiva Milasiene, Olga Belukhina, Remigijus Ivanauskas

**Affiliations:** 1 Faculty of Mechanical Engineering and Design, Department of Production Engineering, Kaunas University of Technology, 51424 Kaunas, Lithuania; olga.belukhina@ktu.edu; 2 Faculty of Chemical Technology, Department of Physical and Inorganic Chemistry, Kaunas University of Technology, 50254 Kaunas, Lithuania; remigijus.ivanauskas@ktu.lt

**Keywords:** synthetic fibers, copper selenide, electrical resistance, cyclic treatment, SEM

## Abstract

Textile production has been steadily increasing for a few decades and, as a result, the amount of industrial textile waste is also increasing. This waste can be reused as raw material to produce new functional composites. Such materials can be used for special purposes with varying combinations of physical and chemical properties by using polymers modified with thin semiconductive or electrically conductive layers of binary inorganic compounds. In this paper, a study of the possibilities of altering the properties of synthetic fiber conductivity by modification with copper selenide is presented. A two-step adsorption/diffusion method was used for the copper selenide layer forming on the surface of the fibers. The treatment process was repeated cyclically. To evaluate the morphological properties of Cu_x_Se treated fibers, scanning electron microscopy (SEM) and energy dispersion X-ray (EDX) were performed. The study showed that the chosen modification method is more suitable for PA and PAN fibers. Dense layers of copper selenides were successfully formed on their surface, which significantly reduced their electrical resistance.

## 1. Introduction

Millions of textile products are created every year around the world, which also affects the amount of waste. The Food and Agriculture Organization (FAO) of the United Nations reported that 3.7 kg of textile fiber per person was consumed in 1950, and in 2007, this figure increased to 11.1 kg of fiber per person [1,2]. The global textile and apparel market is expected to grow at a compound average growth rate of 3.7% per year and exceed 100 million tons by 2025 [3]. Recently, however, it has often been emphasized that textile waste is one of the largest sources of pollution in the world. Therefore, the disposal and management of textile waste is a growing global concern. The EU textile industry generates waste estimated at 16 million tons per year [4]. In response to this problem, researchers face the challenging task of finding a way to use industrial waste as secondary raw material for new applications.

Components of textile waste before consumption are associated with textile raw materials since raw materials for textile production can be divided into three main groups: cellulose fiber, protein fiber, and synthetic fiber [5]. Today, the production of synthetic fibers is greater than that of natural fibers [6]. Thus, synthetic fiber waste is another large group of textile waste whose management, recycling, and reuse issues need to be addressed. The reuse and recycling of textiles could be seen as a way to social and economic benefits and a means of stimulating the nation’s economy [7]. For these reasons, research into the possibilities of using various textile fiber waste to produce new functional materials is becoming increasingly relevant.

Recycled fibers are used as secondary raw materials in very different areas. For example, Sorour Nasimi et al. investigated the possibilities of surface functionalization of recycled polyacrylonitrile fibers with ethylenediamine for highly effective adsorption of Hg(II) ions from contaminated water [8].

Due to the rapidly growing radiation of electromagnetic waves in all spheres of human life, many types of research are being carried out with the purpose to provide various textile materials the electromagnetic field (EMF) shielding properties. Due to this global problem, the development of new materials with effective EMI shielding properties has become a significant direction in science. Zachariah et al., a group of scientists from India, conducted an extensive review of such research. By reviewing more than one-and-a-half hundred articles published in recent decades, they presented a detailed analysis of research of hybrid materials based on polymers, paints, concrete, etc., with diverse fillers for EMF shielding applications. The authors also reviewed materials with different structures, such as fiber-reinforced or aerogel-based polymer composites, foams, various textiles, microfilms, etc., with EMF shielding properties [9].

Researchers are making an effort to look for ways to modify common widely used fibers (PA, PES, PAN, etc.), which are often found in textile waste. Ozen et al. developed nonwoven silver-coated polyamide, Ag/PA66, material using staple fibers with a diameter of 19 (3.3 dtex) and 27 (6.7 dtex). This material has shown excellent weight-normalized specific electromagnetic shielding efficiencies in the range of 1200 dB/(g/cm^3^) in the 0.015–3 GHz range [10]. Wang et al. explored the possibility of modifying the PAN fibers to give them new multifunctional properties. By modifying PAN fibers with (3-aminopropyl) triethoxysilane (APTES) and 3-mercaptopropyltriethoxysilane (MPTES), followed by silver coating, they obtained fibers with antibacterial electromagnetic shielding and antistatic functions. The surface resistance of the modified fibers was obtained to be about 40 mΩ/sq. on average [11].

Chinese scientists Jiang et al. modified PA6 fibers by using a magnetron sputtering system for the deposition of Cu films onto fibers. As a result of this treatment, PA6 fibers received good electrical conductivity. In this study, the influence of winding speed on the thickness of the Cu films was investigated [12].

The Slovenian and English researchers coated the PES fibers with copper using a Pd catalyst and used four polymers to protect the coating AR, PDMS, PUR, and ER. The good conductivity of the samples was demonstrated by the electrical resistance values obtained from the Cu-metalized samples in the range from 0.1 to 1.5 Ω [13].

New special composite materials, with varying combinations of physical and chemical properties, can be obtained by using textile fibers of various nature modified with thin semi-conductive or electrically conductive coatings. Recently, the focus on the possibilities to develop new functional materials using modification with thin layers of a binary chalcogenide, such as copper selenide, has been increasing [14]. Textile materials with semiconductor properties have received broad attention because of their novel electronic, optical, photoelectric, and thermoelectric properties. As an important semiconductor, copper selenide (Cu_x_Se) with nanostructure has potential applications in various fields, such as optical filters, highly efficient solar cells, superionic conductors, electro-optical devices, photothermal conversion, microwave shielding coatings, etc. [15,16].

Companies that produce textile products often have higher or lower levels of yarn waste, which can vary in polymeric nature, such as cellulosic, synthetic protein, or finishing techniques (natural, dated, treated with antibacterial coating). Such waste is usually destroyed, but some companies have recently been involved in the shredding of such waste and the use of its mixtures for the production of insulation materials.

In this study, the research results are part of the investigation of the possibilities of the use of industrial yarn waste as a secondary raw material for the creation of new functional composite materials with EMF shielding properties. Different types of nature knitting yarn waste from Lithuanian companies were studied. In previous research, an electrically conductive copper selenide coating was successfully formed on the surface of wool fibers under the chosen treatment conditions of the adsorption–diffusion method [17].

It is known that synthetic polymers such as polyamide 6 and polyamide 66 have a molecular structure similar to that of proteins. This allows it to be successfully used for the processing of keratin-based solutions into films and nanofibers [18,19]. In addition, because of the presence of several functional groups of amino acid side chains, wool and polyamide fibers have excellent adsorption properties. Additionally, due to similar chemical and physical properties, PA is preferred when mixed with wool when the fabric is to be dyed because PA can be dyed with wool in the same bath. The purpose of this study is to investigate the peculiarities of the Cu_x_Se layer forming on the surface of different synthetic fibers during the cyclic treatment process.

## 2. Materials and Methods

### 2.1. Materials

Four types of synthetic fibers were investigated in this study: 100% polyamide 6 (PA6) (18.00 tex) and 100% polyamide 66 (PA66) yarns (29.00 tex) (W. Barnet GmbH & Co. KG, Aachen, Germany); 100% polyacrylonitrile (PAN) yarns (25.70 tex) (Sunpro Textiles, Gaziantep, Turkey); 100% polyester fibers (PES) yarns (64.50 tex) (W. Barnet GmbH & Co. KG, Aachen, Germany). All of the fiber samples were white in color and 1 m in length.

### 2.2. Treatment Method

**Figure 1:**
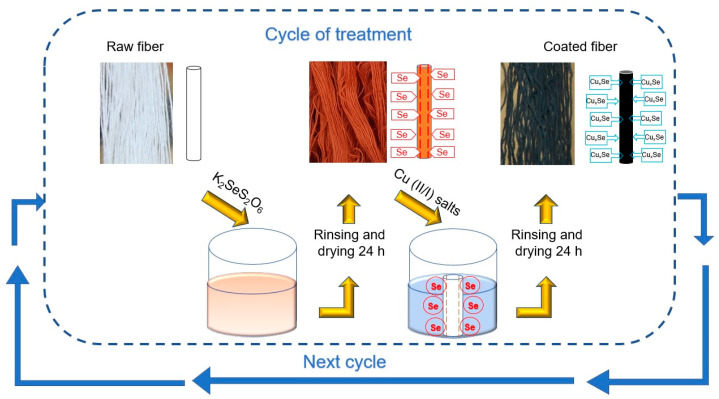
Procedure scheme for the fiber treatment process with Cu_x_Se.

In this study, investigated synthetic fibers were modified with cooper selenides via the two-step adsorption–diffusion method. To explore the possibilities of the use of the mix of different natural industrial fibers waste for the creation of new functional composites, the preparation of treated yarn samples followed the same identical conditions as in previous studies of cyclic modification of wool fibers [17]. One fiber modification treatment cycle consisted of two steps. Each subsequent processing cycle followed the same principle, shown in Figure 1.

In the first stage, the fiber samples were treated with an aqueous solution of K_2_SeS_2_O_6_ (0.1 mol/dm^3^) at pH 2.15 and adjusted with hydrochloric acid. This process occurred at a temperature of 60 °C and takes 90 min. The yarns were then carefully washed with distilled water and dried for no less than 24 h. This surface selenization treatment method has been used successfully in previous studies [19,20]. The preparation procedure of modification materials has been previously reported sequentially [17]. In the second stage, the selenized fibers were treated with Cu (II/I) salts to form Cu_x_Se crystals. The Cu(II/I) salt solution was prepared using crystalline CuSO_4_ 5H_2_O and a hydroquinone reducing agent. In the solution, 85% of copper was in the form of Cu^2+^ and 15% in the form of Cu [20]. The procedure was carried out at 80 °C for 10 min, after which the samples were washed again with distilled water and dried.

### 2.3. Investigative Methods

The mass of the fibers after each treatment cycle was measured with AB104-S Analytical Balance (Mettler-Toledo (Switzerland) GmbH, Greifensee, Switzerland) featuring a measurement range of 110 g ± 0.1 mg, scale interval 0.1 mg, and error (0 ± 0.1) mg. The percentage change in the mass of the samples from the initial mass of the untreated samples was calculated according to the following formula:

∆*m* = ((*m_n_* − *m*_0_)/*m*_0_) × 100%, (1)

where *m*_0_—the mass of an untreated sample (mg); *m*_*n*_—the mass of the sample after the treatment cycle (mg); n—the number of the treatment cycle.

For measuring the electrical resistance of the investigated synthetic fibers, a digital multi-tester PeakTech^®^ 3695 (PeakTech Prüf- und Messtechnik GmbH, Ahrensburg, Germany) (0.1 μA–10 A), a DC Power Supply HY5003 (adjustable voltage 0–50 V), input voltage 50 Hz, and an amperemeter DPM type/model DT9205A (DPMSolid Limited Sp.k., Kowanowko, Poland) were used. Measurements were made constant current of 5 A. During the testing, the fibers samples were clipped between two crocodile connectors of the DMM 20 mm apart and measured 20 times at different locations of the yarns. The electrical resistance was recalculated using the following formula:

*R* = *U*/*I* (Ω), (2)

where *U*—the difference in potential along with the object, V; *I*—the current flowing through the object, A.

A testing machine Zwick/Z005 (sensor KAP-Z 50N) (ZwickRoell GmbH & Co., Ulm, Germany) was used to determine the tensile properties of the investigated fibers. The experiment was carried out according to the EN ISO 2062:2009 [21]. The experimental length of the samples was 500 mm, and the tensile speed was 500 mm/min. The test of each yarn variant was repeated with 50 samples.

Scanning electron microscopy (SEM) was performed using the Quanta 200 FEG (FEI CompantTM, Eindhoven, The Netherlands) to investigate the surface morphology of treated fibers. The SEM image was taken in the energy range of 10 kW and 30 kW at a 0-degree tilt angle of the samples, a magnification of 5000 times, and a scale of 20 µm. Synthetic fiber samples were visualized at a residual pressure of 80 Pa, sufficient to avoid image artifacts, i.e., sample charging, which usually occurs when analyzing a high-energy electron beam. The samples were not additionally processed for SEM and EDX testing. For energy dispersion X-ray (EDX) imaging, the QUANTAX EDS system with a Bruker XFlash^®^ 4030 detector and ESPRIT software (Bruker AXS Microanalysis GmbH, Berlin, Germany) was used.

## 3. Results

### 3.1. Physical Measurements

**Figure 2:**
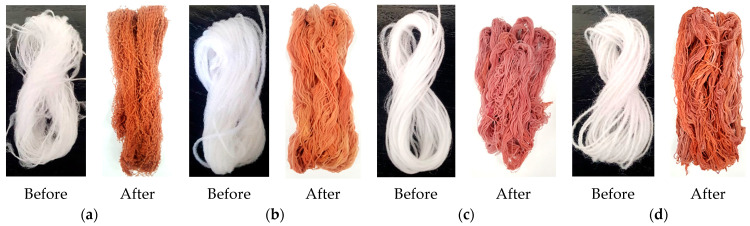
Examples of synthetic fibers after the first stage of selenium: (**a**) PA6, (**b**) PA66, (**c**) PAN, and (**d**) PES.

Observation of color changes in chemically processed specimens is often used in studies to identify a successful modification process. To facilitate monitoring of fiber changes during the treatment process, all investigated synthetic fibers were selected for the study in white. The visible change in color of all types of synthetic fibers from white to reddish after the first stage of seleniumization showed the successful formation of secondary ions such as SeSO_3_^2−^ and Se_2_S_2_O_6_^2−^ on the surface of the investigated samples (Figure 2). This result was an important first sign that the selected conditions of treatment are suitable for previously successfully modified wool as well as for investigated synthetic fibers.

**Figure 3:**
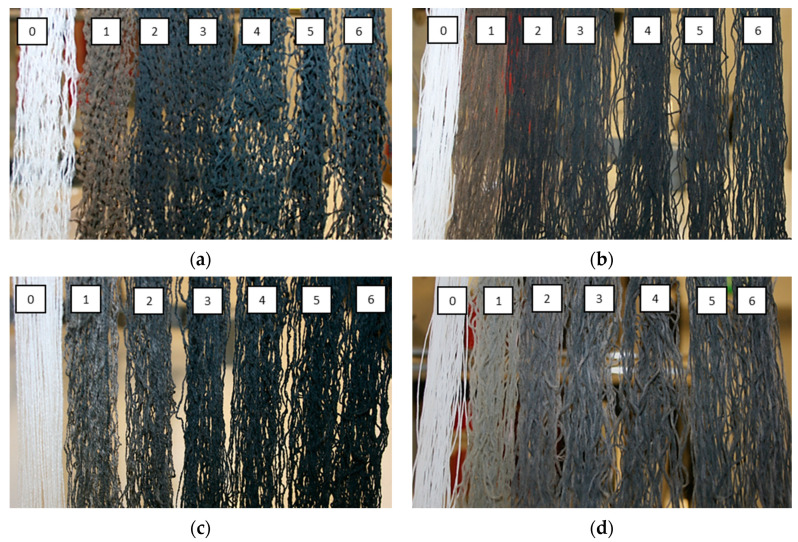
Example of modified synthetic fibers from left to right range from raw to treated after 6 cycles: (**a**) PA6, (**b**) PA66, (**c**) PAN, and (**d**) PES.

The change in color during the second treatment step from reddish to gray shows that the seleniumized synthetic fibers reacted with the Cu(I/II) ion solution and indicates the formation of Cu_x_Se crystals on their surface (Figure 3). By repeating the modification cycles, the coating layer of Cu_x_Se thickened, and the fiber samples became increasingly blacker. In the case of PA6, PA66, and PAN fibers, samples after the fourth modification cycle became completely black. On the other hand, it was observed that the PES fiber samples remained dark gray even after six treatment cycles. This leads to the suspicion that the layer of copper selenide crystals was worse-formed than in the case of other fibers.

In materials science research, to confirm the fact of surface modification effectivity, the SEM analysis of the material surface and determining the chemical composition of formed coating are most commonly used. However, in fast pilot experiments or in search of optimal process conditions, and especially in the cycle modification process, it is appropriate to observe the weight gain of the modified samples. The gain in textile mass as an indicator of the coating process is often used by researchers in the development of functional materials with various coatings [22,23,24,25,26,27]. The growth of the copper selenide crystal coating layer on the surface of the investigated wool fibers was first judged by the weight gain of the samples [17].

**Figure 4:**
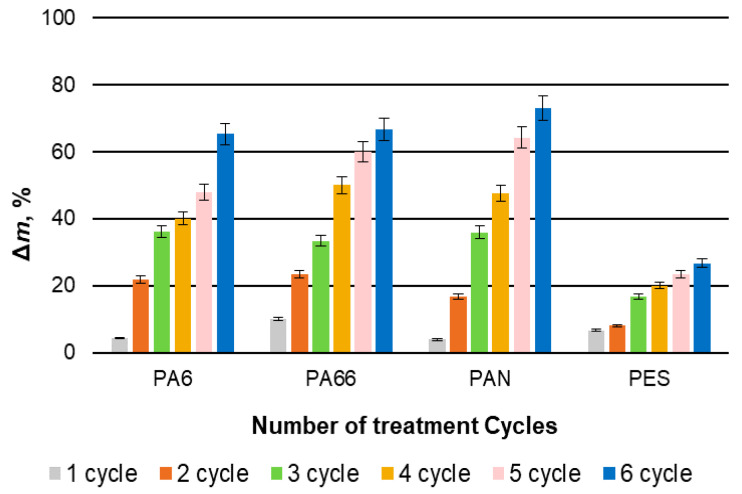
Variation in the mass of the investigated synthetic fibers during the process of modification with copper selenide.

As can be seen in Figure 4, in the case of investigated PA6, PA66, and PAN fibers, after each treatment cycle, the mass growth was obvious, and after six treatment cycles, it reached more than 60%. On the other hand, such results are lower than in the case of protein fibers, when ∆m after six treatment cycles was approximately 180% [17]. It is also obvious that the mass growth of treated PES fibers clearly increased less, and after six treatment cycles, it did not reach 30%.

**Figure 5:**
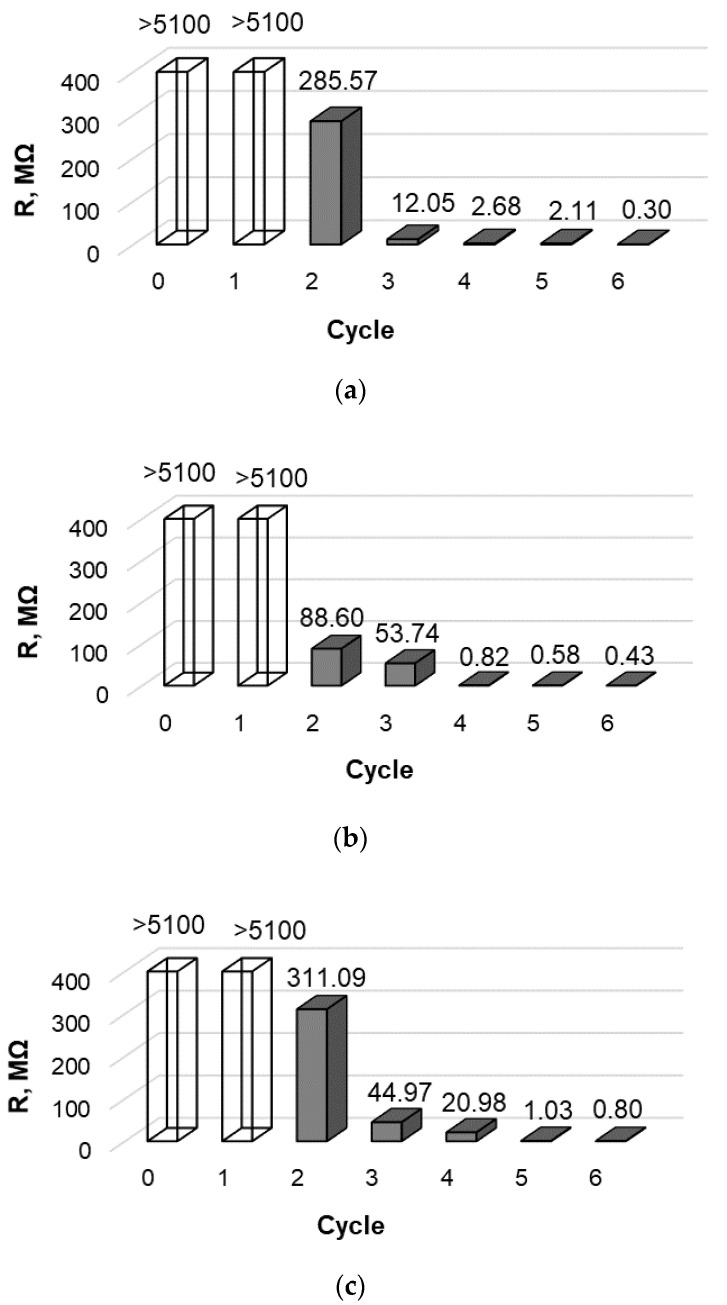
Distribution of resistance measurement with synthetic fibers: (**a**) PA6, (**b**) PA66, and (**c**) PAN.

Before treatment, all investigated synthetic fibers were dielectric materials, so the electrical resistance of both untreated fibers and samples after the first treatment cycle was greater than 5100 MΩ; therefore, it could not be measured with the equipment used in this research. For all fibers, the situation remained unchanged after the first treatment cycle; however, the second repeat of the treatment cycle already significantly reduced the electrical resistivity of PA6, PA66, and PAN fiber samples to the measured value (Figure 5). By repeating the treatment cycles, the electrical resistance of these three fibers continued to decrease and less than 1000 KΩ was measured after six cycles. It was determined that, after the fourth cycle, the intensity of the increase in electrical conductivity of the investigated synthetic fibers decreased.

All samples showed the highest relative errors for the electrical resistance measurement after the second treatment cycle when the resistance decreased to a measurable value. In this case, values of resistance of PA6 were 258.57 ± 25.71 MΩ, respectively; PA66 values were 88.60 ± 4.47 MΩ, and the PAN—311.09 ± 22.28 MΩ. This is due to the fact that, after the first cycles, the coating of copper selenide crystals had not yet formed as a solid layer. After each subsequent cycle, the crystal coating thickened, and the random error of the values of electrical resistance of all types of investigated fibers decreased and was less than 5%.

The slight increase in mass of only about 25% and the gray color of the modified samples indicated that the copper selenide layer formed on the surface of the PES fiber may be insufficiently dense and homogeneous. Electrical resistance measurements confirmed this, as even after six modification cycles, the resistance of the PES fiber samples did not fall below the measurable limit.

### 3.2. Tensile Properties Analysis

The results of the study of mass grown and the electrical resistance of the investigated synthetic fibers showed that the higher number of modifying cycles increased the coating quality of the formed copper selenide crystal. However, by improving the quality of the copper selenide layer, the duration of aggressive chemical treatment also increased, which can adversely affect the structure of the fiber itself.

To analyze this effect, the influence of the modification process on the mechanical properties of the coated fibers was investigated. The results of the performed standard tensile test are shown in Table 1, Table 2, Table 3 and Table 4.

**Figure 6:**
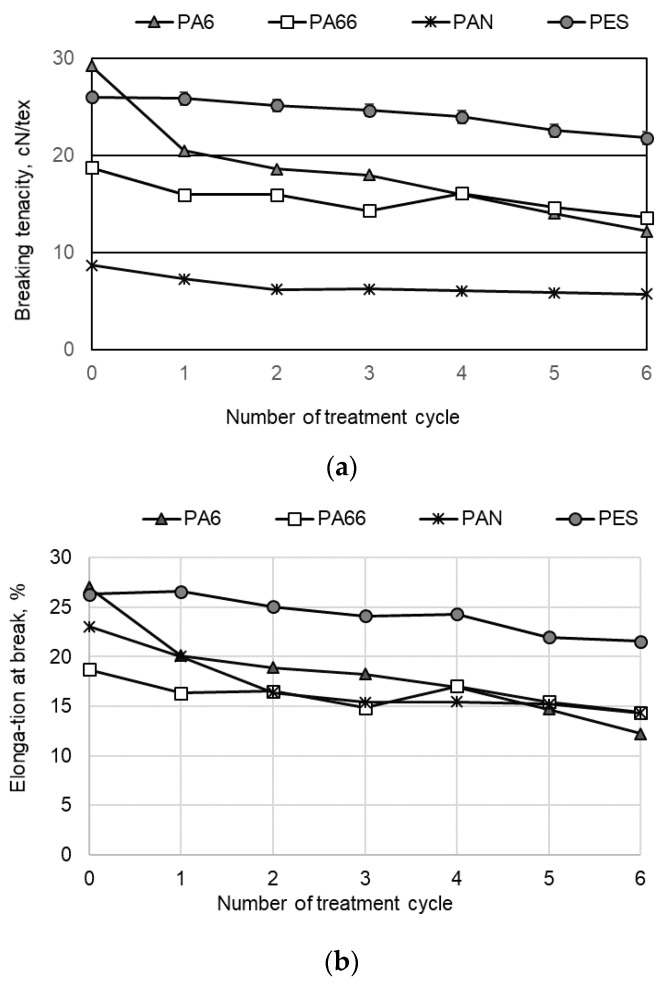
Influence of the number of the treatment cycle on the (**a**) breaking tenacity and (**b**) the elongation at break of investigated synthetic fibers.

It was found that the influence of the treatment process on the tensile properties results differed in the cases of PA6 and PA66: the average values of breaking tenacity of PA66 decreased by about 27%, and elongation at break decreased by about 23%, while the results of PA6 decreased about 58% and 54%. The corresponding results for PAN fibers were 34% and 38% (Figure 6).

As can be seen from the obtained results, the treatment process least changed the tensile properties of the investigated PES fibers. This effect is due to the different chemical nature of the polyester material. It is clear that the reagents used for the treatment procedure and the modification conditions did not sufficiently affect the PES fiber’s surface; therefore, the modified yarns did not lose their strength.

### 3.3. SEM and EDX Analysis

**Figure 7:**
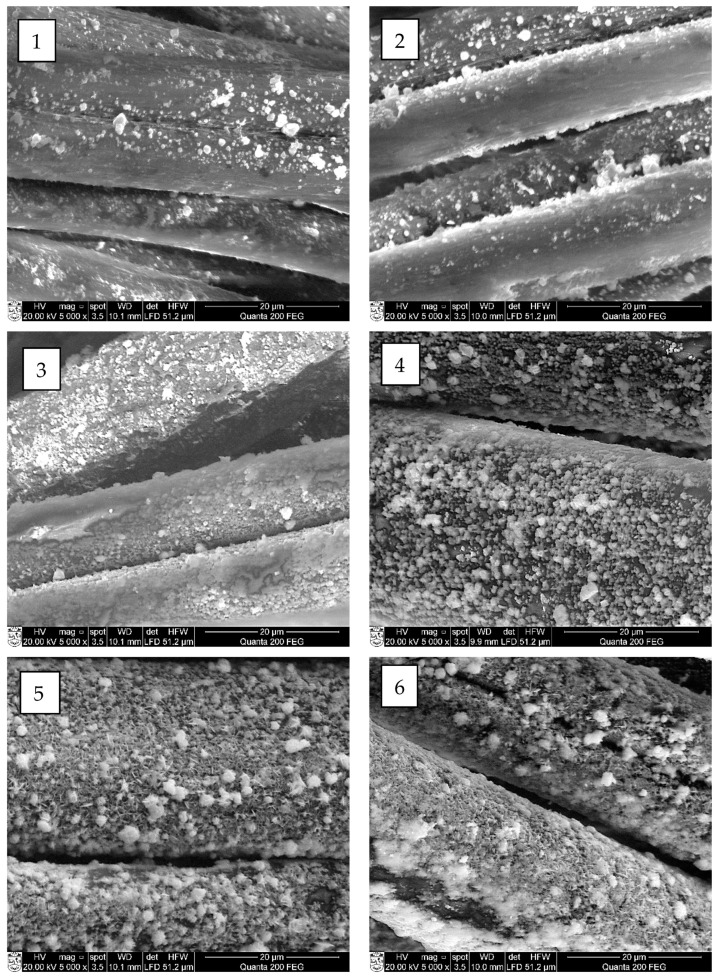
SEM images of copper selenide-coated PAN fibers after different numbers of treatment cycles: (**1**) first (mag. 5000×); (**2**) second (mag. 5000×); (**3**) third (mag. 5000×); (**4**) fourth (mag. 5000×); (**5**) fifth (mag. 5000×); and (**6**) sixth (mag. 5000×).

**Figure 8:**
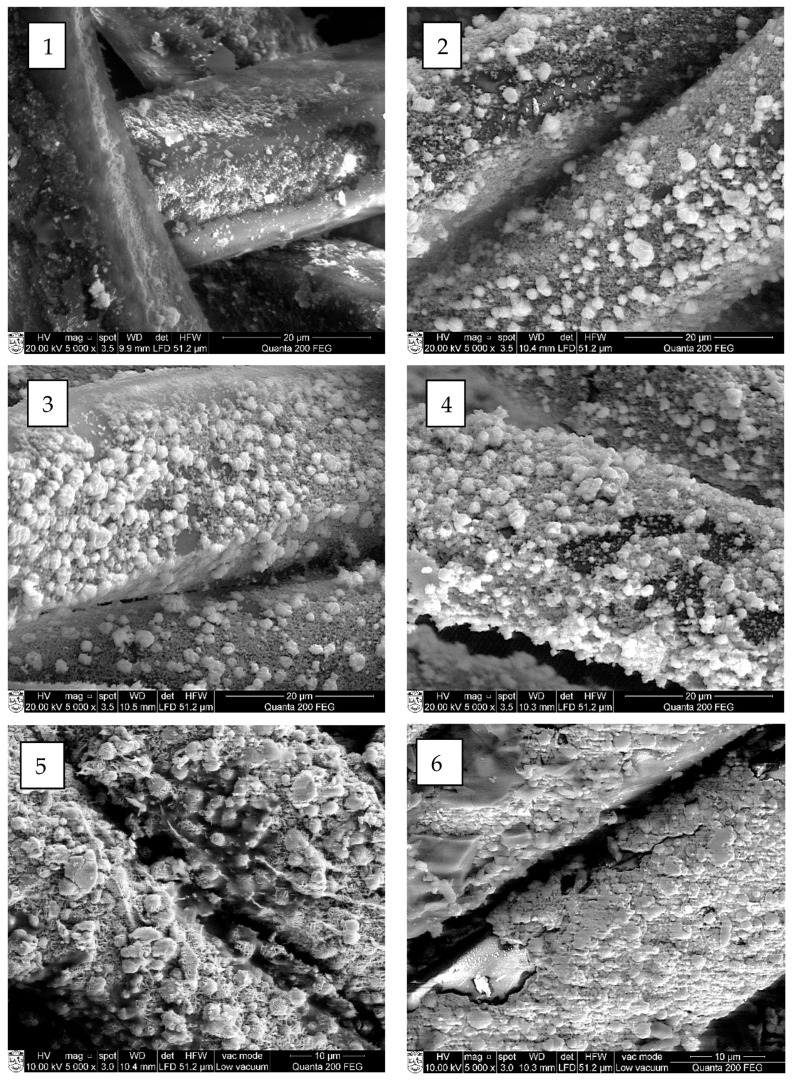
SEM images of copper selenide-coated PA6 fibers after different numbers of treatment cycles: (**1**) first (mag. 5000×); (**2**) second (mag. 5000×); (**3**) third (mag. 5000×); (**4**) fourth (mag. 5000×); (**5**) fifth (mag. 5000×); and (**6**) sixth (mag. 5000×).

**Figure 9:**
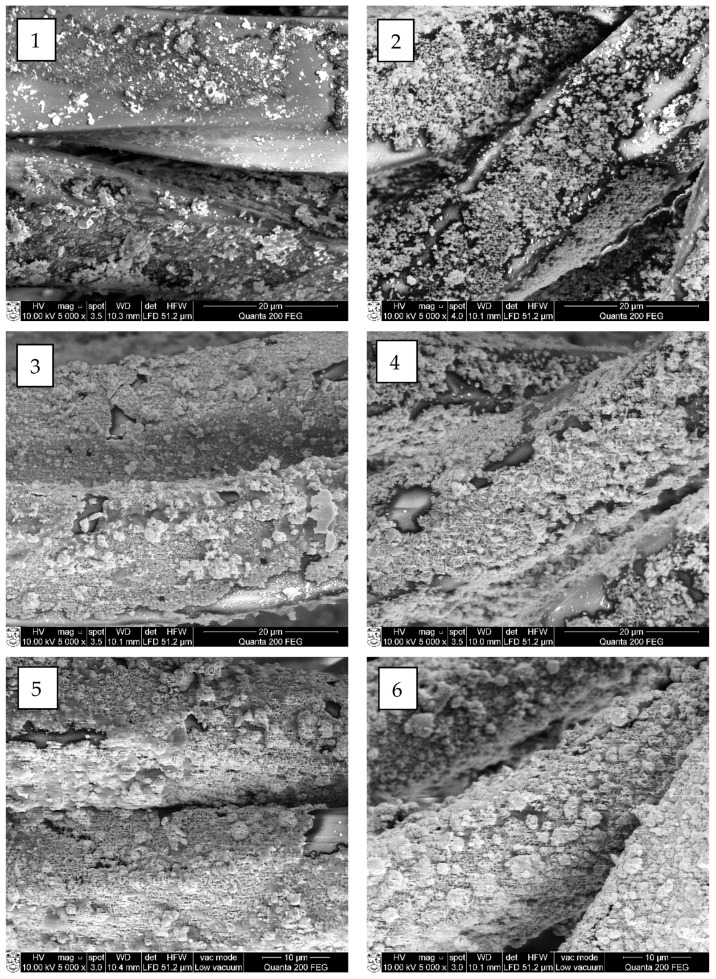
SEM images of copper selenide-coated PA66 fibers after different numbers of treatment cycles: (**1**) first (mag. 5000×); (**2**) second (mag. 5000×); (**3**) third (mag. 5000×); (**4**) fourth (mag. 5000×); (**5**) fifth (mag. 5000×); and (**6**) sixth (mag. 5000×).

**Figure 10:**
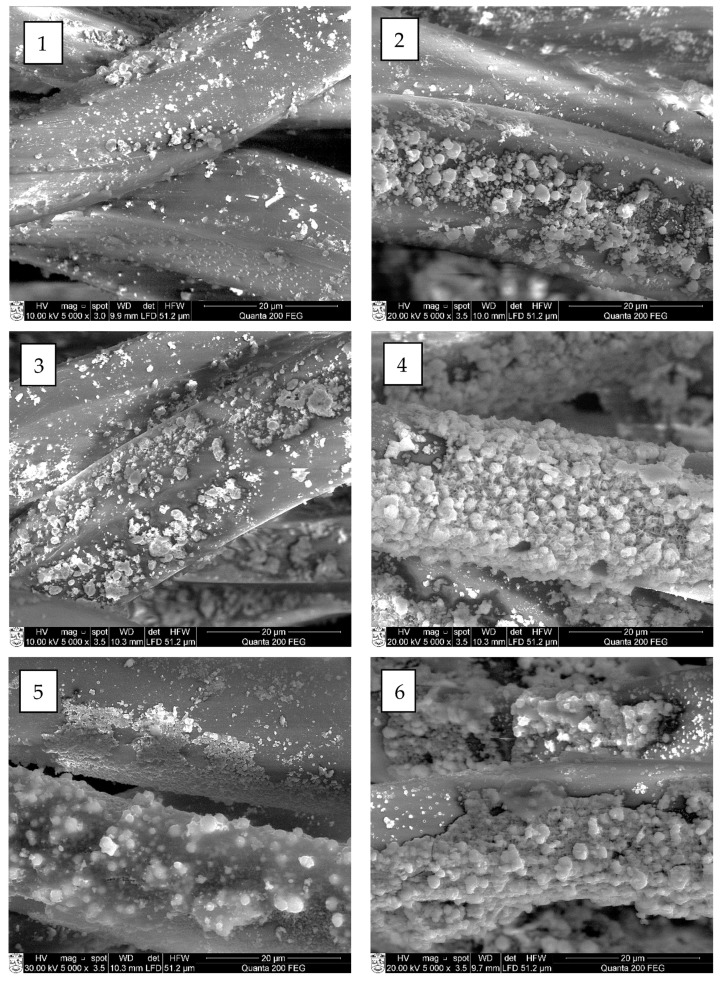
SEM images of copper selenide-coated PES fibers after different numbers of treatment cycles: (**1**) first (mag. 5000×); (**2**) second (mag. 5000×); (**3**) third (mag. 5000×); (**4**) fourth (mag. 5000×); (**5**) fifth (mag. 5000×); and (**6**) sixth (mag. 5000×).

The SEM and EDX analyses were carried out to estimate the influence of chosen cycle treatment conditions on the quality of cooper selenide coating on the surface of treated fibers. Performed SEM images of investigated fibers are presented in Figure 7, Figure 8, Figure 9 and Figure 10 (“mag.”—magnification).

The SEM examination showed that the first crystal agglomerates had already formed on the surface of all investigated synthetic fibers after the first treatment cycle. As can be seen, the surface of all types of treated fiber filaments was not yet covered with a continuous layer of copper selenide crystals.

At first, on the surface of modified fiber filaments, smaller Cu_x_Se crystals agglomerated, which, after further cycles, grew and expanded until they filled the entire surface. The multiplication of copper selenide crystals, most of which were very small, less than 1 µm in diameter, was already visible on the surface of all four different fibers after the first cycle (Figure 6, Figure 7(1), Figure 8(1), Figure 9(1) and Figure 10(1)). After the second and third treatment cycles, the surface of PA6, PA66, and PAN fiber filaments was almost completely covered with a continuous layer of copper selenides (Figure 6, Figure 7(2,3), Figure 8(2,3) and Figure 9(2,3)). As the modification cycles continued, the copper selenide coating thickened; therefore, the fiber mass increased, and electrical resistance decreased.

It should be noted that a low-quality, discontinuous, cracked, and unmeasurable electrical resistance Cu_x_Se coating was formed on the surface of the PES fibers. The reason for this is due to the synthetic fibers used in the study—the surface of PES fibers is the most hydrophobic, so the fibers have the least water absorption capacity. This results in low permeation flux due to hydrophobic interactions between the surface of the fibers and the solute in the original solution. As a consequence, the adhesion between the formed Cu_x_Se layers and the surface of the PES fibers is weak, which greatly complicates the formation of high-quality layers. This explains the formation of lower quality Cu_x_Se layers on the surface of the fibers of this polymer [28].

In summarizing the results of the study on the formation of Cu_x_Se layers on the surface of synthetic fibers, it can be noted that it did not grow as a uniform film. First, during the first cycle, crystallization centers were formed on the surface of the fibers, on which crystals of Cu_x_Se and their agglomerates grew. As the number of cycles increased, the agglomerates combined to form a continuous coating on the surface of the fibers.

Furthermore, a significant influence of the origin of the fiber and the number of treatment cycles on the morphology of the Cu_x_Se coating was clearly seen.

**Figure 11:**
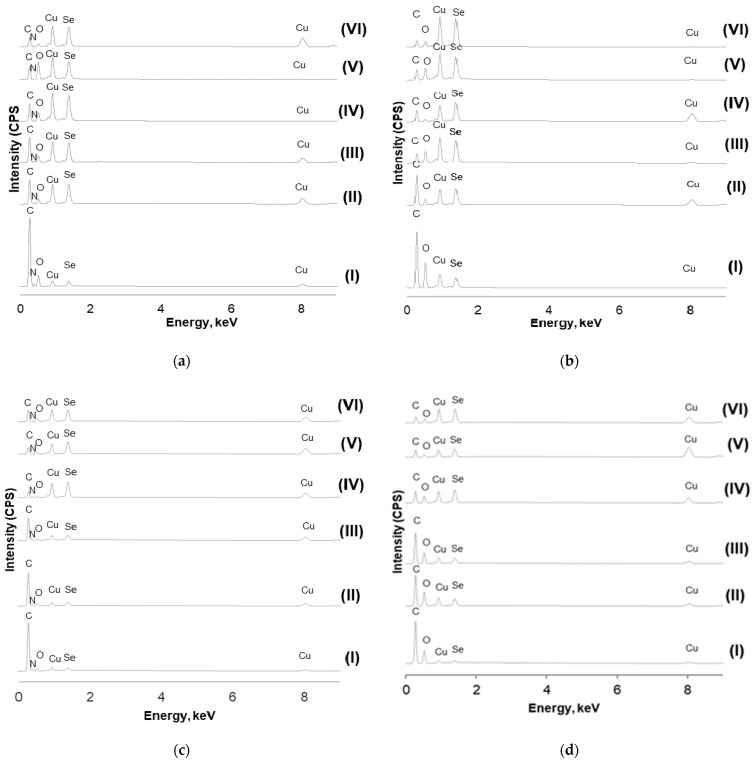
EDX spectra of treated synthetic samples after different numbers of cycles: (**a**) PA6, (**b**) PA66, (**c**) PAN, and (**d**) PES.

Based on the data obtained and the results of the EDX analysis, several remarks can be made. First of all, the dominant peaks were associated with strong C, O, and N signals in synthetic fibers, as well as with complex Cu and Se X-ray lines (Figure 11).

**Figure 12:**
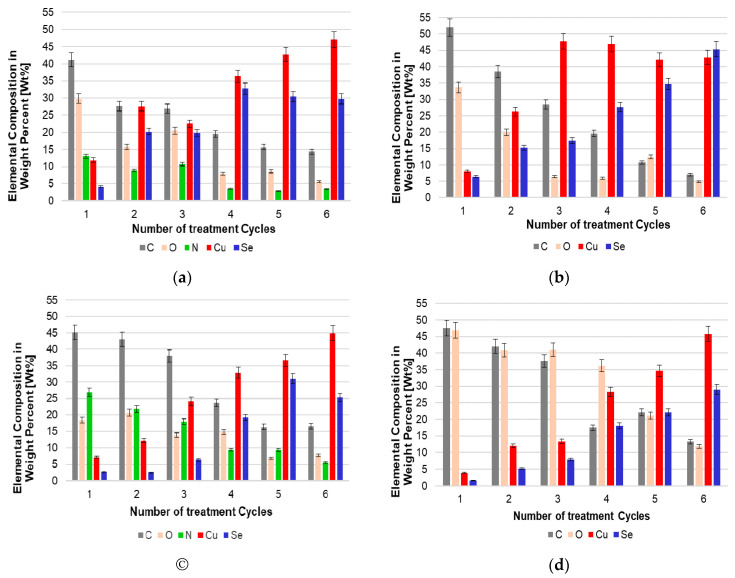
Surface elemental composition of the investigated treated fibers: (**a**) PA6, (**b**) PA66, (**c**) PAN, and (**d**) PES.

Other investigators [29,30,31] also reported peaks corresponding to C, O, and N obtained from the analysis of untreated PAN fibers, PA6 fibers [32], PA66 [33], and PES fibers [34]. The Cu and Se peaks emanating from the Cu_x_Se coatings confirmed the formation of a well-defined thin coating layer. Another important observation is that, after each subsequent processing cycle, the intensity of the Cu signals at 0.907 and 8.04 eV and the Se signal at 1.38 eV gradually increased, while the intensities of the C, N, and O signal peaks, respectively, at 0.297 keV, 0.360 keV, and 0.485 eV, gradually decreased (Figure 11). The dynamics of a monotonous increase in the content of copper and selenium, with a decrease in the concentration of other elements, are clearly visible from the diagrams presented in Figure 12. These results clearly indicated that, after each subsequent cycle, the Cu_x_Se layer on the fibers became thicker and denser. 

These results confirmed the impression based on the visual analysis of SEM images.

## 4. Conclusions

Currently, one of the great challenges for researchers is finding ways to use significant amounts of textile waste for new purposes as secondary raw materials. In this study, the possibilities of altering the electrical conductivity of industrial waste of synthetic polymer yarn for knitted products were examined. For the investigated synthetic fibers modification with cooper selenide thin layers, the two-stage adsorption–diffusion method was used. The treatment process was carried out cyclically and was repeated six times. Studies have shown that such conditions are appropriate for the successful modification of the surface of PA6, PA66, and PAN fibers.

The electrical resistance of the nonconductive fibers (before treatment > 5100 MΩ) already decreased to the measurable values after the second cycle of modification. After a series of six cycles, the resistance of all three mentioned fibers decreased and was less than 1000 KΩ.

The SEM and EDX analysis confirmed the formation of a layer of copper selenide crystals on the surface of the investigated synthetic fibers. 

It is clear that the reagents used for the treatment procedure and the modification conditions did not sufficiently affect the surface of the PES fiber; therefore, the modified yarns did not lose their strength. Performed experiments showed that choused reagents and treatment procedures did not sufficiently affect investigated polyester fibers (PES). The SEM analysis confirmed that the cooper selenide layer on the surface of this polymer composition was not sufficiently dense and homogeneous.

Such resistance of PES fibers to a sufficiently aggressive modification medium and a small reduction in the mechanical tensile properties may be useful for a mix of different-nature fiber waste that is better covered by Cu_x_Se but is strongly weakened due to chemical damage.

## Figures and Tables

**Table 1:** Tensile characteristics of investigated PA6 fibers.

Characteristics		Treatment Cycle Number						
		0	1	2	3	4	5	6
Breaking tenacity, cN/tex	Min and max value, cN/tex	27.23/30.79	10.75/23.52	6.19/21.72	6.62/22.96	8.87/19.73	3.42/18.84	2.70/22.12
	X *, cN/tex	29.25	20.49	18.61	18.02	16.04	14.05	12.19
	Δ, cN/tex	±0.35	±0.73	±0.71	±0.79	±0.71	±1.75	±1.71
	Relative error, %	1.20	3.58	3.82	6.20	5.40	12.43	14.05
Elongation at break, %	Min and max value, %	24.00/28.70	10.47/23.20	6.15/22.62	7.14/24.87	9.44/20.85	2.77/19.82	2.02/21.32
	X, %	27.04	20.10	18.88	18.23	16.95	14.68	12.22
	Δ, cN/tex	±0.43	±0.77	±0.74	±0.85	±0.81	±1.88	±1.77
	Relative error, %	1.59	3.87	3.94	6.24	5.63	12.82	14.50

* X—average (arithmetic mean).

**Table 2:** Tensile characteristics of investigated PA66 fibers.

Characteristics		Treatment Cycle Number						
		0	1	2	3	4	5	6
Breaking tenacity, cN/tex	Min and max value, cN/tex	18.14/19.62	7.81/18.23	8.41/18.19	5.72/17.23	13.82/18.16	6.89/18.21	5.61/17.42
	X *, cN/tex	18.78	15.98	15.97	14.34	16.14	14.65	13.60
	Δ, cN/tex	±0.17	±0.69	±0.41	±0.74	±0.31	±0.70	±0.81
	Relative error, %	0.91	4.33	2.55	5.17	1.94	4.77	5.93
Elongation at break, %	Min and max value, %	17.92/19.49	7.41/19.43	8.26/18.66	5.31/18.16	14.22/19.01	7.17/19.39	5.90/18.60
	X, %	18.70	16.34	16.54	14.82	17.04	15.43	14.36
	Δ, cN/tex	±0.26	±0.81	±0.48	±0.83	±0.40	±0.83	±0.8
	Relative error, %	1.41	4.97	2.90	5.59	2.32	5.39	6.13

* X—average (arithmetic mean).

**Table 3:** Tensile characteristics of investigated PAN fibers.

Characteristics		Treatment Cycle Number						
		0	1	2	3	4	5	6
Breaking tenacity, cN/tex	Min and max value, cN/tex	7.61/9.69	5.89/8.49	4.86/7.87	4.57/7.70	4.49/7.60	4.23/7.94	4.46/7.19
	X *, cN/tex	8.68	7.30	6.20	6.25	6.07	5.89	5.74
	Δ, cN/tex	±0.23	±0.18	±0.17	±0.22	±0.17	±0.18	±0.17
	Relative error, %	2.71	2.48	2.89	3.58	2.95	3.21	3.09
Elongation at break, %	Min and max value, %	20.85/24.75	16.60/22.28	12.98/19.73	11.08/18.90	11.23/18.92	10.62/18.90	10.93/17.03
	X, %	23.04	20.00	16.40	15.41	15.46	15.22	14.32
	Δ, cN/tex	±0.42	±0.39	±0.42	±0.50	±0.41	±0.43	±0.42
	Relative error, %	1.83	1.97	2.57	3.26	2.66	2.86	2.94

* X—average (arithmetic mean).

**Table 4:** Tensile characteristics of investigated PES fibers.

Characteristics		Treatment Cycle Number						
		0	1	2	3	4	5	6
Breaking tenacity, cN/tex	Min and max value, cN/tex	24.35/27.42	23.54/27.81	20.23/27.43	15.96/27.65	19.27/26.74	13.05/24.81	17.08/25.34
	X *, cN/tex	26.01	25.88	25.17	23.99	24.68	21.82	22.58
	Δ, cN/tex	±0.32	±0.28	±0.41	±0.56	±0.50	±0.64	±0.57
	Relative error, %	1.26	1.09	1.66	2.36	2.06	2.97	2.53
Elongation at break, %	Min and max value, %	23.55/28.46	22.63/30.16	18.65/28.63	16.85/29.41	16.33/29.16	12.40/27.20	14.86/25.30
	X, %	26.31	26.58	25.07	24.12	24.30	22.00	21.58
	Δ, cN/tex	±0.55	±0.52	±0.59	±0.77	±0.82	±0.79	±0.72
	Relative error, %	2.09	1.99	2.38	3.22	3.40	3.61	3.36

* X—average (arithmetic mean).

## Data Availability

The data presented in this study are available on request from the corresponding author.
